# Social negotiation and “accents” in Western lowland gorillas’ gestural communication

**DOI:** 10.1038/s41598-024-75238-y

**Published:** 2024-10-28

**Authors:** Jacques Prieur, Katja Liebal, Simone Pika

**Affiliations:** 1 Comparative Developmental Psychology, Department of Education and Psychology, Freie Universität Berlin, Habelschwerdter Allee 34, Berlin, 14195 Germany; 2 Human Biology and Primate Cognition, Institute of Biology, Leipzig University, Talstrasse 33, Leipzig, 04103 Germany; 3 Cognitive BioCognition, Institute of Cognitive Science, Faculty of Human Sciences, University of Osnabrück, Artilleriestrasse 34, Osnabrück, 49076 Germany; 4 Department of Education and Psychology, Comparative Developmental Psychology, Freie Universität Berlin, Habelschwerdter Allee 45, Berlin, 14195 Germany

**Keywords:** Social behaviour, Evolutionary developmental biology

## Abstract

Recent findings on chimpanzee infants’ gestural development show that they use some gesture types flexibly and adjust them depending on their interaction partner and social context, suggesting that gestural communication is partly learnt and partly genetically determined. However, how gesture types are shaped by social and demographic factors remains unclear. We addressed this question by focusing on gesture type morphology and conducted a fined-grained analysis of gestural form during intraspecific social-play interactions in two captive groups of Western lowland gorillas (*Gorilla gorilla gorilla*). We focused on the most frequent gesture types (beat chest, slap body, slap ground and touch body) produced by subadults (infants, juveniles and adolescents). We considered twelve morphological gesture characteristics (e.g., horizontal and vertical hand trajectories, fingers flexion and spread). Our multifactorial investigation shows that morphological characteristics of distinct gesture types can be shaped by social factors, namely signaller’s sociodemographic characteristics (group and kinship), signaller’s behavioural characteristics (body posture) and context-related characteristics (recipient’s sex, attentional state and position in the signaller’s visual field). We nurtured the lively debate concerning gesture origins by revealing the existence of “accents” in non-verbal communication and the highly variable adjustment of gestural form to different conspecifics and interactional characteristics, which supports the revised social negotiation hypothesis.

**Supplementary Information:**

The online version contains supplementary material available at 10.1038/s41598-024-75238-y.

## Introduction

Human and other animals, particularly our closest living relatives, the non-human primates (hereafter primates), engage in complex communication^1–3^. Recently, a lot of research attention has been focusing on the acquisition of gestures^4–8^. Three non-mutually exclusive mechanisms have been postulated aiming to explain the acquisition and development of great apes’ gestural signalling: phylogenetic ritualisation, ontogenetic ritualisation, and social negotiation^5,8–10^.

Phylogenetic ritualisation is a process in which a communicative signal is postulated to have evolved from a functional action sequence that initially lacked a communicative function (e.g., the dominance signal “mounting”). This means that signals are ‘borrowed’ from other contexts (e.g., a sexual context^10,11^). Some researchers suggested that evidence for phylogenetic ritualisation has been provided by studies on gestural signalling of gorillas (three captive, one wild group^12^), chimpanzees (one community in the wild^9^), and bonobos (two neighbouring groups in the wild^13^). For instance, these authors reported that a very small amount of gesture types of chimpanzees (two out of 66 distinct gesture types^7,9,14^) and gorillas (eight out of a 100 gesture types^7,12,15,16^) are species typical as a result of genetical channelling. Moreover, they found that 36 gesture types overlapped between the four genera in the great ape family (bonobos, chimpanzees, gorillas, and orangutans)^7^. They suggested that a part of great apes’ gestural production is innate but they exhibit flexibility in their usage across contexts. One criticism of the phylogenetic ritualisation hypothesis is, however, that it overlooks gestural production with regards to signaller (e.g., social factors such as kinship, hierarchy and affiliation), and context-related characteristics (e.g., recipient’s sociodemographic factors and audience effect such as recipient’s attentional state) as well as recipient affordances (see^8,17^ for more details about the limitations of this hypothesis).

The ontogenetic ritualisation hypothesis proposes that a communicative signal originates from two individuals shaping each other’s behaviour in repeated instances of an interaction over time^18^. For instance, Tomasello writes that ‘play-hitting is an important part of the rough-and-tumble play of chimpanzees, and so many individuals develop a stylized ‘arm-raise’ to indicate that they are about to hit the other and thus initiate play’^19^. Tomasello and his colleagues argued that the role of ontogenetic ritualisation for gestural acquisition has been shown by several studies^5,20^. For instance, investigations of the Leipzig Gesture group focused on the size and variability of gestural repertoires of all four great ape species (chimpanzees, bonobos, gorillas, orangutans; two groups each) and one smaller ape species (siamangs *Hylobates syndactylus*, four captive groups; see^18^for more details). They showed the existence of idiosyncratic gestures (i.e. gestures that are used by single individuals only within a group and are possibly learned by individual learning) and high degrees of variability within and between groups for chimpanzees and bonobos but not for gorillas^21–24^. The researchers suggested that in contrast to imitative learning, great apes learn their gestures via repeated interactional exchanges between two individuals. Nevertheless, there are several limitations questioning the validity of the ontogenetic ritualisation hypothesis^7,8,25,26^). For instance, two studies categorizing chimpanzees’ and gorillas’ gestural repertoires^9,12^were not able to identify mechanically effective sequences of manual actions (theoretically deprived of a communicative function but see^27–29^for a different opinion) that are assumed to become ritualized into a gesture^30^. Moreover, gestures acquired via ontogenetic ritualisation within dyads are not assumed to be generalized across dyads^7,31,32^, imposing high costs on learners^8,33^. For instance, if this acquisition route would be true each individual would need to invest a considerable amount of time and energy to acquire a gestural repertoire that is understood by most of its group members and conversely to also understand the meaning of conspecifics’ gestures directed towards itself.

A third proposed pathway to gestural acquisition is learning via social negotiation, a process based on the assumption that an already existing action can be used and function as a communicative signal^8,33–36^. Function and use of actions and gestures can be characterized by a four-dimensional continuum that reflects fine modulation of behavioural expression in relation to the social environment^27^, suggesting that their morphological features can be similar, sometimes mechanically effective and sometimes mechanically ineffective, directed or non-directed and inducing or not a voluntary response. The revised social negotiation hypothesis (*sensu*^35,37^) by Fröhlich and Pika^8,33^posits that gestures originate from repeated exchanges of social behaviours (more or less mechanically effective, directed and response-inducing) between interactants, resulting in a shared understanding that certain behaviours can be used as communicative signals to convey distinct information associated with particular social contexts (e.g., play, travel) to attain desired goals. Interactants also learn that particular social partners, characterized, among other things by their respective age, sex, group, hierarchical status and ties of kinship and affiliation, can assign different meaning/s to gestures resulting in different outcomes. Contrary to the ontogenetic ritualisation process, acquired knowledge within a given dyad can be generalized among dyadic relationships within the group^8^. So far, first evidence supporting the idea that recipients’ attributes influence gestural production stem from a systematic analysis of gestural development in infant chimpanzees (*Pan troglodytes verus*; *Pan troglodytes schweinfurthii*) living in two communities in their natural environments^33,38,39^. The authors showed that infant chimpanzees adjusted their gestural play solicitations in relation to specific attributes of conspecifics such as the age, sex and kin relationship^38^. Moreover, they reported that depending on the infants’ age, gesture frequency, gesture production in sequences, and sizes of the gestural repertoires increased in dyads of non-maternal conspecifics with higher communicative interaction rates and in relation to the number of previous interaction partners. In contrast, communicative interaction rates with mothers did not impact on these aspects of infant chimpanzees’ gestural signalling^39^. The authors concluded that gestural acquisition and development of gestures, at least in infant chimpanzees, does not result from the shortening of a functional action sequence (*sensu *ontogenetic ritualisation)^38^. Rather, gestural interactions are mutual online adjustments (unlike phylogenetic ritualisation) and are shaped via repeated exchanges by both interactants^26,40^. Hence, gestures arise through learning via social negotiation, and can be flexibly used and adjusted across contexts and therefore also vary in form^7^.

Research on primate gestural communication has mostly focused on the usage and social function of gestures^4,14,18,21,41^while considerably less studies used a form-based approach^42–44^. Furthermore, these few studies focusing on gesture form have not investigated if and how gesture form is linked to sociodemographic factors of the two interacting individuals. Taking such an approach would enable a more detailed understanding of the possible influence of these socioecological factors on gestural development and which mechanisms underlie their acquisition. Thus, if we assume that gestures are largely innate as suggested by Byrne and colleagues^7,9,14^, this does not necessarily mean that they cannot be flexibly used, as signallers can learn to adapt their gesture usage to the attentional state of the recipient or context of interaction. However, we would expect little variation in gesture form across individuals (e.g. no effect of group nor social context would emerge on gestural form), as signallers should be less capable of changing morphological aspects of their gestures. This would correspond to the usage of vocalizations, which are largely innate: while their usage can be adjusted, nonhuman primates are less likely to change structural aspects of their vocalizations^40,45^. If ontogenetic ritualisation is the mechanism underlying gesture acquisition, we would expect gesture form to vary across dyads (e.g. effect of group but not of recipient’s attentional state and position in relation to signaller would emerge on gestural form). It seems highly unlikely given the effort necessary to shape dyad specific repertoires and which so far has not been found in apes^46,47^. Therefore, we study the morphology of gorilla gestures within the framework of the social negotiation hypothesis, suggesting that gestures are modified depending on the social context and the characteristics of the interactants.

Here, we aimed to address the question if and how demographic and social factors influence gestural form in gorillas. We investigated gestural production during spontaneous intraspecific social interactions in two captive groups of gorillas, with a special focus on dyadic social-play interactions initiated by subadults (infants, juveniles and adolescents)^48,49^. We particularly focused on a specific communicative function — initiation of play-fighting — for the following two reasons: (1) Play-fighting is a crucial context for the development of cognitive, psychological, and social skills of many species, including humans^50–52^, and (2) it represents a communicative niche involving frequent gestural solicitations to initiate and end social play in gorillas^12,22^. In addition, by keeping the behavioural outcome (i.e. social play-fighting) constant this approach enabled us to investigate gestures carrying the same meaning^38,39^. More specifically, we focused on four of the most frequently produced gesture types by gorillas (i.e. beat chest, slap body, slap ground and touch body; from here on gestures are depicted in small capitals)^22,53^. For each gesture type, we considered twelve gesture characteristics (i.e. manuality, manual laterality, gesture target, hand position in relation to signaller’s body, horizontal hand trajectory, vertical hand trajectory, main moving body part, physical contact with the recipient, thumb and fingers flexion as well as thumb and fingers spread) at the stroke phase, which is functionally the most meaningful phase of a gesture^54,55^. Gorillas are specifically relevant models for this study for three reasons:

- first, they exhibit the largest gestural repertoire of all non-human great apes in terms of overall number and number per individual: 33–102 gesture types^12,18^,
- second, the gorilla social structure is consistent with many features of human social organisation such as certain patterns of parental behaviours (e.g. male parenting, family formation)^56^. For example, both gorilla parents provide offspring care (in the wild^57^: in captivity: personal observation) contrary to the other non-human great ape species (i.e. chimpanzees, bonobos, and orangutans) for whom maternal care is largely predominant and paternal care is rare or negligible^58^.
- third, the gorillas differ from the very much studied chimpanzees in their ecology and their social structure and dynamics: chimpanzees are both terrestrial and arboreal^59^and live in multi-male–multi-female groups characterized by a highly variable party membership, whereas gorillas are mainly terrestrial^60^and live in polygamous and generally cohesive groups^61^. Studying gorillas’ acquisition and development of gestural communication may thus provide crucial insight in how human development, communicative and social-cognitive skills are linked and related to social environment.

We applied a multifactorial approach taking simultaneously into account three categories of factors: signaller’s sociodemographic characteristics (age, sex, group and kinship), signaller’s behavioural characteristics (signaller’s body position and motion) and characteristics related to the context of signal production (recipient’s age and sex, recipient’s attentional state, position of the recipient in the signaller’s visual field and interindividual proximity). In line with the revised social negotiation hypothesis^7^and previous research (see^4^ for a recent review), we predicted that gorilla signallers’ gestural form would be particularly modulated by signaller’s sociodemographic characteristics, characteristics related to the context of signal production and, to a lesser extent, by signaller’s behavioural characteristics. Notably, we expected gesture type morphology differences between groups. If we find social pressure effects on gesture type morphology caused by sociodemographic characteristics (e.g. kinship relatedness and group belonging) and social context characteristics (e.g. recipient’s attentional state and position of the recipient in the signaller’s visual field), this will support the revised social negotiation hypothesis, but not the phylogenetic and ontogenetic ritualisation hypotheses.

## Results

### Overview of the gestural data set

We recorded a total of 662 gestures produced by 14 subadults during play-fighting interactions: 161 beat chest, 151 slap body, 166 slap ground and 184 touch body (interaction distribution: 605 subadult–subadult and 57 subadult–adult interactions). On average, each of our 14 study subadults contributed 47.3 ± 35.6 (mean ± SD, Apenheul = 45.4 ± 43.9, Burgers = 49.8 ± 24.1) gesture occurrences to this data set.

### Factors influencing morphological characteristics of gestures

For each of the four gesture types, we focused on signaller’s sociodemographic, signaller’s behavioural and context-related characteristics to investigate factors influencing signaller’s morphological characteristics of gestures. For each gesture type and associated occurrences, we carried out twelve GLMM analyses taking successively into account each of the twelve dependent variables (e.g., fingers flexion, hand vertical trajectory) (see Electronic Supplementary Table 1 for a descriptive summary of dependent, fixed and random variables). The best GLMM model was determined by backward stepwise comparison. The analysis of deviance results corresponding to each best GLMM model with significant results are displayed in Table 1. For clarity, only significant and trend significant p-values of post hoc multiple comparison tests are presented in the paragraph below whereas all p-values of each best GLMM model with significant results are presented in the Appendix Table A1. For each gesture type, several of the twelve GLMM analyses did not show any significant results or the associated fitted models were singular (i.e., the parameters were on the boundary of the feasible parameter space: variances of one or more linear combinations of effects were (close to) zero; the corresponding analyses are not presented here.

**Table 1:** Analysis of deviance table (type II Wald Chi-square tests).

Gesture type	Dependent variable	Fixed variables	χ^2^	Df	*P*
Beat chest	Fingers spread	Zoo	7.631	1	**0.006**
		R_location	2.792	1	0.095
	Main moving body part	S_sex	0.820	1	0.365
		Zoo	0.104	1	0.747
		R_attention	6.054	2	**0.048**
		R_location	1.951	1	0.162
	Manuality	R_location	0.424	1	0.515
		Posture	8.285	3	**0.040**
	Manual laterality	R_sex	9.864	1	**0.002**
		Posture	6.099	3	0.107
		Bodymotion	3.797	2	0.150
	Vertical hand trajectory	S_sex	1.972	1	0.160
		Zoo	10.984	1	**0.001**
		R_attention	6.752	2	**0.034**
		Posture	24.126	3	**2.35e-05**
Slap body	Manuality	R_age_classe	2.661	3	0.447
		Zoo	4.019	1	**0.045**
		R_attention	2.267	2	0.322
		Bodymotion	0.383	2	0.826
	Hand location	R_sex	3.333	1	0.068
Slap ground	Manuality	Kinship	5.730	2	0.057
		S_age_classe	3.370	2	0.185
		R_attention	2.210	2	0.331
		R_location	3.658	1	0.056
		Posture	24.566	6	**4.11e-04**
	Manual laterality	R_attention	5.600	2	0.061
		Posture	6.099	6	0.412
		Bodymotion	0.489	2	0.783
	Hand location	R_sex	5.402	1	**0.020**
		R_attention	5.671	2	0.059
		R_location	0.546	1	0.460
		Bodymotion	2.194	2	0.334
	Vertical hand trajectory	Zoo	15.820	1	**6.97e-05**
		R_attention	3.586	2	0.166
		Posture	15.247	6	**0.018**
Touch body	Manual laterality	Zoo	0.938	1	0.333
		R_attention	7.085	2	**0.029**
		R_location	2.932	1	0.087
		Posture	8.079	6	0.232
	Gesture target (Head/Lowerbody)	R_attention	10.707	2	**0.005**
		R_location	0.843	1	0.359
	Gesture target (Lowerbody/Upper body)	R_attention	9.334	2	**0.009**
		R_location	0.012	1	0.914

Table 1 shows the analysis of deviance (Type II Wald chi-square tests) corresponding to each best GLMM model with significant results, for each of the four gesture types. χ^2^: value of the type II Wald chisquare; Df: Degree of freedom; P: p-value of the type II Wald chisquare. Significant results are in bold.

#### Beat chest

Our results concerning beat chest showed that signaller’s sociodemographic characteristics (group), signaller’s behavioural characteristics (body posture) and context-related characteristics (recipient’s sex and attentional state) influenced its following five morphological characteristics differently: finger spread, main moving body part, manuality, manual laterality and vertical hand trajectory (Table 2).

**Table 2:** Results of post hoc multiple comparisons tests.

Gesture type	Dependent variable	Findings
Beat chest	Fingers spread	S used half-spread fingers more than bonded fingers at Burgers’zoo / at Apenheul (*p* = 0.006)
	Main moving body part	S used their elbow more than their wrist when the recipient faced the signaller / when the recipient was half attended (*p* = 0.040)
	Manuality	S used bimanual gestures more than unimanual gestures when standing bipedally / when lying on their back (*p* = 0.048)
		S tended to use bimanual gestures more when sitting / when lying on their back (*p* = 0.068)
	Manual laterality	S used their right hand more than their left hand when the recipient was a female / a male (*p* = 0.002)
	Vertical trajectory	S used an up-to-down trajectory more than a down-to-up trajectory at Apenheul / at Burgers’zoo (*p* = 0.001)
		S used an up-to-down trajectory more when the recipient faced the signaller / when the recipient was half attended (*p* = 0.029)
		S used a down-to-up trajectory more than an up-to-down trajectory when standing bipedally / when lying on their back (*p* = 0.015) or sitting (*p* = 0.0001)
		S tended to use an up-to-down trajectory more when sitting / when standing tripedally (*p* = 0.051)
Slap body	Manuality	S used bimanual gestures more than unimanual gestures at Apenheul / at Burgers’zoo (*p* = 0.045)
	Hand location	S tended to place their hand far from their body more than placing their hand between body midline and sides of the body when the recipient was a male / a female (*p* = 0.068)
Slap ground	Manuality	S used bimanual gestures more than unimanual gestures when signalling towards half-siblings / siblings (*p* = 0.049)
		S used bimanual gestures more when standing bipedally / when standing tripedally (*p* = 0.001) or when lying on their front (*p* = 0.015)
		S tended to use bimanual gestures more when standing bipedally / when sitting (*p* = 0.077) and more when sitting / standing tripedally (*p* = 0.077)
		S tended to use bimanual gestures more when the recipient was in their left visual field during an interaction / their right visual field (*p* = 0.056)
	Manual laterality	S tended to use their right hand more than their left hand when the recipient faced the signaller / when the recipient was half attended (*p* = 0.056)
	Hand location	S placed their hand far from their body more than placing their hand between body midline and sides of the body when the recipient was a male / a female (*p* = 0.020)
		S tended to place their hand far from their body more than between body midline and sides of the body when the recipient faced the signaller / when the recipient turned its back to the signaller (*p* = 0.086)
	Vertical trajectory	S used an up-to-down trajectory more than a down-to-up trajectory at Apenheul / at Burgers’ zoo (*p* = 0.0001)
		S used an up-to-down trajectory more when sitting (*p* = 0.011) or lying on their front (*p* = 0.024) / when using “other body positions”
		S tended to use an up-to-down trajectory more when standing tripedally / when using “other body positions” (*p* = 0.083)
Touch body	Manual laterality	S used their right hand more than their left hand when the recipient faced the signaller / when the recipient was half attended (*p* = 0.035)
		S tended to use their right hand more when the recipient turned its back to the signaller / when the recipient was half attended (*p* = 0.060)
		S tended to use their right hand more when the recipient was in their right visual field during an interaction / their left visual field (*p* = 0.087)
	Target type (Head vs. Lowerbody)	S touched the recipient’s head (*p* = 0.004) or upper body part (*p* = 0.008) more than the recipient’s lower body part when the recipient faced the signaller / when the recipient turned its back to the signaller

Table 2 shows the factors influencing morphological characteristics of gestures. S: Signallers; /: compared to; Half attended: the direction of the recipient’s head was turned at 90° in relation to the direction of the signaller’s head.

#### Slap body

Our findings concerning slap body indicated that signaller’s sociodemographic characteristics (group) and context-related characteristics (recipient’s sex) influenced its following two morphological characteristics differently: manuality and hand location (Table 2).

#### Slap ground

Our results concerning slap ground revealed that signaller’s sociodemographic characteristics (group and kinship), signaller’s behavioural characteristics (body posture) and context-related characteristics (recipient’s sex, attentional state and position in the signaller’s visual field) influenced its following four morphological characteristics differently: manuality, manual laterality, hand location and vertical hand trajectory (Table 2).

#### Touch body

Our findings concerning touch body demonstrated that context-related characteristics (recipient’s attentional state and position in the signaller’s visual field) influenced its following two morphological characteristics differently: manual laterality and gesture target (Table 2).

## Discussion

The aim of the present study was to test specific aspects of the revised social negotiation hypothesis^7,33^. More specifically, we investigated whether demographic and social factors influence gestural form. To address this question, we investigated communicative interactions of subadult gorillas, with a special focus on four frequently used gesture types to initiate play fighting (i.e. beat chest, slap ground, slap body and touch body). Overall, our results showed that signaller’s sociodemographic characteristics (group and kinship), signaller’s behavioural characteristics (body posture) and context-related characteristics (recipient’s sex, attentional state and position in the signaller’s visual field) influenced the form of the four study gesture types differently. On the contrary, our results did not reveal any significant influence of signaller’s age, sex and body motion as well as recipient’s age on gestural form. These findings therefore shed new light on gestural acquisition and development since the few gestural form studies in both human and nonhuman animals only paid attention to the influence of communicative function (e.g., pointingin humans^62,63^:; touchin chimpanzees^43^; push and hand-onin gorillas^26,64^, signaller’s age (infants versus juveniles, subadults or adults^64^; infants versus adults^43^) or to gesture characteristics (pointing frequency, accompanying vocalizations, and mothers’ pointingin humans^65^). In the following paragraphs, we will discuss our results in more detail and with regards to the revised social negotiation hypothesis^7^. Furthermore, and due to the present findings not allowing for direct comparisons with the existing literature on gestural form, we will discuss our results in light of the more extensive vocal form literature^66–71^.

### Influence of signaller’s sociodemographic characteristics (group and kinship)

Our fine-grained analysis suggests a group effect concerning specific morphological gestural characteristics in gorillas. For instance, we found that the subadult gorillas at Burgers’ zoo produced beat chest more often using half-spread fingers than bonded fingers compared to at Apenheul. When producing beat chest or slap ground, signallers at Apenheul used more often an up-to-down trajectory than a down-to-up trajectory than the studied individuals at Burgers’ zoo. When performing slap body, signallers at Apenheul used bimanual gestures more often than unimanual gestures compared to the individuals at Burgers’ zoo. There may be different ecological, genetic and social explanations. Following the method of exclusion^72–74^and its criticism by Laland and Janik^75^, we ruled out the ecological and genetic variations explanations as the main processes that have shaped the production of beat chest, slap ground and slap bodyin our study gorillas because (1) the two neighbouring Dutch zoos considered in our study have the advantage of housing western lowland gorillas under very similar environmental conditions and (2) there are no systematic genetic differences between the two captive social groups (see^76^ for more details). Therefore, it seems more likely that social group pressure might be the main processes that have shaped the production of beat chest, slap ground and slap bodyin our study gorillas, which is in line with our predictions. In non-human animals, studies suggest that social pressure plays a key role in the evolution of communication, for instance eliciting dialect forms in the vocalization patterns of many taxa, mainly primates^69,77–80^, birds^81–83^and cetaceans^84–88^. In humans, within-spoken language variation at any linguistic level (i.e. from phonology to syntax) is shaped by multiple intertwined factors such as the speakers’ geographical location, their socio-demographic characteristics (e.g., gender, age, ethnicity) and the interactional context of speech (e.g., the level of formality, the recipient’s identity) (^89^; see^67,90^ for reviews). Here, we showed variations in the morphological characteristics of three shared gesture types (beat chest, slap ground and slap body) between two distinct social groups of the same subspecies hence, a difference in the way these three gesture types are shaped or pronounced. Within a comparative approach, we deliberately qualified such variations as “accents” defined in the linguistic literature as the way in which speakers from different geographic locations (e.g., England and Scotland) produce differently a shared signal type (e.g., the word “bird”) in terms of quality of voice, pronunciation, distinction of vowels and consonants, stress, and prosody^91^(see^92^ for the application of this definition in non-human animal acoustic research). We hypothesize that not only gorillas but also other animal species including humans may show “accents” in their gestural communication but this remains to be confirmed by further fined-grained analysis.

Considering the influence of kinship ties between signallers and recipients, we found that subadult gorillas performed slap ground more using two hands than one hand when communicating towards half-siblings compared to siblings. It might be less easy to initiate play with half-siblings than with siblings who are more socially bonded and who are potentially more used to interact between each other, as showed in wild chimpanzees^39^. It might thus be useful to attract half-siblings using two hands that amplify the auditory and visual component of this particular gesture. As far as we know, there have been no primate studies yet investigating the influence of kinship on gestural or vocal form so this is difficult to draw comparisons. In a broader comparative approach, our observed modulation of gestural form in relation to the genetic relatedness of the playing partners is in accordance with a recent study of wild-derived house mice (*Mus musculus musculus*) showing that vocal form depended upon genetic relatedness of potential mating partners: males emitted longer and more complex ultrasonic vocalizations towards unrelated females than related females^93^. Social rank status and the quality of relationships (i.e. affiliation) have been shown to influence the use of gestural, vocal, facial expression and gaze signals in primates^70,94–96^; see^4^ for a recent review). For instance, wild mountain gorillas’ close calls are correlated with the social rank status of an individual: dominant signallers produce more syllabled calls, particularly *double grunts*, whereas subordinate signallers produce more nonsyllabled calls such as *grumbles* and *hums*^97,98^. In chimpanzees, subordinate signallers used their right-hand less for gestures towards a strong than towards a medium affiliative subordinate partner^94^. We hypothesized that psychosocial stress effects (that would inhibit the right hemisphere and thus could elicit a right-side bias at the population level as supported by several studies in human and non-human primates; e.g., see reviews^99,100^ for more details) would be less important when subordinates interact with other subordinates and particularly during interactions involving pairs of strong affiliative partners.

### Influence of signaller’s behavioural characteristics (body posture)

The impact of posture on vocal form has been studied in great detail across decades in human communication^68,101–104^. Several researchers showed that body posture interferes with larynx position, respiratory function and vocal tract shape, thereby modulating vocal production and performance. As far as we know, however, there have been no studies yet investigating the influence of posture on gestural form.

We found that the signaller’s body posture influenced morphological characteristics of the two auditory gestures (beat chest and slap ground) but not the two tactile gestures (slap body and touch body). When performing beat chest, subadult gorillas used bimanual gestures more often than unimanual gestures when standing bipedally compared to when lying on their back. Signallers were very likely to perform beat chest with both hands when standing bipedally, holding their hands below their chest before gesturing (personal observations), possibly to better stabilize their bipedal posture by keeping their centre of gravity as low as possible. These constraints on bipedal balance control imply a subsequent down-to-up hand trajectory to beat the chest. This would explain why we found that signallers producing beat chest used a down-to-up trajectory more than an up-to-down trajectory when standing bipedally compared to when lying on their back or sitting. This tended also to be true when they were standing tripedally rather than sitting.

When performing slap ground, signallers preferred to use bimanual gestures when standing bipedally than unimanual gestures compared to when standing tripedally or lying on their front. Physical constraints associated with bipedal posture would explain such differences. Indeed, gorilla signallers standing bipedally most often used both hands instead of one hand to keep stability when slapping (vigorously) a surface to avoid impacting it with their upper body (personal observations). Using both hands amplifies the auditory and visual components of this particular gesture and this might help to attract even more the attention of the recipient who can better hear and see it. Further studies analyzing the influence of signaller’s gestural forms on the recipient’s response could test this hypothesis.

### Influence of context-related characteristics (recipient’s sex, recipient’s attentional state and position of the recipient in the signaller’s visual field)

We found a sex difference concerning the performance of the gesture beat chest: subadult individuals across groups performed the gesture more often with their right hand than their left hand when the recipient was a female compared to a male. These results are in line with previous data on chimpanzees (pooled data considering both subadult and adult chimpanzees and auditory, tactile and visual gestures as a whole^94^. However, they contradict previous observations collected on gorillas in the zoos of Apenheul and Burgers (The Netherlands) and La Vallée des Singes (France) showing no effect of the recipients’ sex on handedness (pooled data considering both subadult and adult gorillas and auditory, tactile and visual gestures as a whole^28^).

Our results also indicated that signallers producing slap ground placed their hand far from their body more often than placing their hand between body midline and sides of the body when the recipient was a male compared to a female. This tended also to be true for slap body. Young human males and subadult male rhesus monkeys perform more vigorous and rougher physically active social play than females^105–107^. Based on personal observations, we hypothesized that this was also the case for our study subadult gorillas and that placing the hand farther from the signaller’s body might be a defensive way to protect the signaller itself. So far, there have been no primate studies investigating a recipient’s sex effect on signal form (e.g. on gestural shape or the amplitude of vocalizations). Our finding showing variation of gestural form in relation to the recipient’s sex is in accordance with a recent study of wild-derived house mice^108^. The authors found a trend toward higher amplitudes of ultrasonic vocalizations by males when presented with a male compared to a female conspecific.

Considering the influence of the recipient’s attentional state, the results showed that signallers performed beat chest more often using their elbow as main moving body part than their wrist when the recipient faced the signaller compared to when the recipient was half attended. They also used an up-to-down trajectory more often than a down-to-up trajectory to perform beat chestwhen the recipient faced the signaller than when the recipient was half attending. It may be possible that signallers do so to amplify the visual component of the gesture when the recipient is attending in order to attract them even more to play. This explanation is in line with recent findings on all four non-human great apes and some monkey species demonstrating that all four non-human great apes and some monkey species are able to adjust their gestural use to the recipients’ attentional state, so that a signaller produces gestures more towards a recipient oriented towards it or uses the adequate type of gesture (chimpanzees^24,30,71^; bonobos^21,109^; gorillas^12,22,109^; orangutans^23,110,111^; olive baboons^112,113^; red-capped mangabeys^114^).

When performing slap ground or touch body, signallers used their right hand more often than their left hand when the recipient faced the signaller compared to when the recipient was half attended. This finding suggests that an audience effect (i.e., a change in behaviour caused by being observed by another person) induces signallers’ right-hand use. An audience effect might lead to a certain level of arousal (stress)^115,116^ that would inhibit the right hemisphere and thus increase right-hand use as mentioned above.

In addition, we found that signallers performing touch body touched the recipient’s head or upper body part more than its lower body part when the recipient faced the signaller compared to when the recipient turned its back to the signaller. Actually, the recipient was more likely to stand quadrupedally when turning its back to the signaller (personal observations). Therefore, the lower body part of the recipient was more easily reachable for the signaller than the recipient’s head or upper body part, which could explain the present finding. To our knowledge, there is no study investigating the effect of the recipient’s attentional state on vocal form in primates and other taxa. However, there are studies investigating the usage of vocalizations depending on the recipient’s attentional state in chimpanzees^71,117,118^. For instance, captive chimpanzees produced a higher number of calls and non-vocal attention-getting behaviors to attract the human experimenter to get a food reward when the latter was facing away from, compared to facing towards the chimpanzee^71^. Conversely, chimpanzees used more different visual signals when the human experimenter human was oriented toward them.

Considering the influence of the position of the recipient in the signaller’s visual field, signallers performing slap groundtended to use bimanual gestures more than unimanual gestures when the recipient was in their left visual field during an interaction (SVF_L) compared to their right visual field (SVF_R). The laterality literature shows a dominance of the right hemisphere (associated with the left eye/visual field) for the recognition of face and emotional facial expressions in non-human primates^119,120^. When the recipient was in the signaller’s left visual field (right hemisphere in control) during an interaction (SVF_L), the signaller’s arousal/emotional state might be higher and would more likely drive production of a bimanual gesture than a unimanual gesture. So far, there is no study of the influence of the position of the recipient in the signaller’s visual field on vocal form in primates and other mammals.

### Testing the revised social negotiation hypothesis in detail

So far, studies on the acquisition of gestures in primates provided evidence for modulations of gesture use in relation to signaller’s sociodemographic characteristics (age, sex and group, kinship relatedness of the playing partners) and context-related characteristics (behavioural context, interaction rates and maternal proximity) (captive gorillas^26^; wild chimpanzees^38,39^). Here, we add another facet to this literature by showing that morphological characteristics of gestures produced by signallers can also be modulated in relation to particular signaller’s sociodemographic and behavioural characteristics (e.g., kinship relatedness with its playing partners and signaller’s body posture) and to context-related characteristics (e.g., recipient’s attentional state). These findings are thus in accordance with our prediction: the revised social negotiation hypothesis (*sensu*^35,37^) postulating that gestural use and form show flexible adjustment to signallers’ sociodemographic characteristics and social context characteristics is fully supported for the gestural modality^7^. On the contrary, our findings are not in line with the phylogenetic and ontogenetic ritualisation hypotheses. Together, the above-mentioned vocalization studies, particularly in humans^66–68^and chimpanzees^69–71^indicated that the use and form of specific vocalizations (e.g. differences between the English and Scottish word accents in humans^66^; differences in the acoustic structure of pant hoots of male chimpanzees living in two neighbouring communities in the Taï forest, Côte d’Ivoire^69^) also show flexible adjustment to individual sociodemographic matrices and interactional circumstances. The revised social negotiation hypothesis thus appears also to be supported for the vocal modality, at least in humans and our closest living relatives, the chimpanzees. Importantly, however, we do not claim that learning via social negotiation is the only mechanism involved in the acquisition and development of great apes’ gestural and vocal communication systems and, more broadly speaking, primates’ communication signalling (Cf^4^). Interestingly, the human and non-human primate literature focusing on facial expressions and gaze shows that their usage could be flexible according to sociodemographic characteristics of the interactants and interactional characteristics^4^ but what about their form? In depth-investigations are needed to fill in the gaps and nurture the literature on verbal and non-verbal communicative signalling form and associated functions.

## Conclusion

Applying an unprecedented and fine-grained methodology, we provide the first evidence that subtle morphological characteristics of several distinct gesture types can be shaped by demographic, social, behavioural and context-related factors. Among other things, we discovered that accents can be found in non-verbal communication signals! Our findings strengthen the fact that gestural production of signallers can exhibit a highly variable online adjustment in relation to interactants and context-related characteristics^26,38,39,121^. The revised social negotiation hypothesis^7^ postulating that gestures show flexibility in their usage and their form was previously supported for gesture use, it is now also verified for gesture form. The strength of this study is that many interactions per gesture type were analysed by four observers in total with good levels of agreement between them. However, we emphasize the limitation of generalizing these conclusions as we only considered two social groups of gorillas.

Disentangling the complex relationship between genetic and socio-ecological factors that influence signal acquisition and development in human and non-human animals represents a fascinating challenge. To meet this challenge and to shed more light on the evolutionary roots of human language, further comparative and multifactorial investigations taking into account (1) a variety of communicative signalling forms of distinct signal types (e.g., distinct facial expression types), (2) additional signaller’s sociodemographic characteristics (e.g., signaller’s hierarchical status and affiliation), signaller’s behavioural characteristics (e.g., the signaller’s facial and gazing behaviours) and context-related characteristics (e.g., behavioural context and emotional valence), (3) the recipient’s response to the signaller and (4) other animal populations and species living in different social and ecological niches are needed.

## Methods

### Individuals

Twenty-five western lowland gorillas raised under semi-natural conditions were observed at two zoos: Apenheul Primate Park and Burgers’ Zoo in the Netherlands. The age categories of individuals were based on Breuer and colleagues’^48^definitions for infants (0–3 years old), juveniles (4–6 years old) and adolescent individuals (7–11 years old) and on Stoinski and colleagues’^49^ categories for young (12–20 years old) and mature (> 20 years old) adult individuals (see Table 3). At the time of the data collection in 2017, the gorillas (14 subadults: 2 infants, 8 juveniles and 4 adolescents; 11 adults: 6 young adults, 5 mature adults; 15 females and 10 males) ranged in age from 0.3 to 46 years (Mean = 12.08; SD = 11.18). For a detailed description of the morphology, ecology, social structure, organization, behaviour and housing conditions of the gorillas see^76^.

**Table 3:** Individual characteristics of the study group of gorillas.

Name	Age (years)	Sex	Zoo
*Mature adults (over 20 years)*			
Mintha	43	F	Apenheul
Mandji	42	F	Apenheul
Bauwi	28	M	Burgers
N’Gayla	24	F	Burgers
Jambo	23	M	Apenheul
*Young adults (12–20 years)*			
Kisiwa	20	F	Apenheul
Nimba	18	F	Burgers
Nemsi	16	F	Apenheul
Gyasi	15	F	Apenheul
Makoua	13	F	Burgers
N’Aika	12	F	Burgers
*Adolescents (7–11 years)*			
N’Akouh	7	M	Burgers
Wimbe	9	M	Apenheul
Mapasa	9	M	Apenheul
Mfungaji	8	F	Apenheul
*Juveniles (4–6 years)*			
Mzungu	6	M	Apenheul
Chama	6	F	Apenheul
Tayari	6	F	Apenheul
Iriki	6	F	Apenheul
Jabari	4	M	Apenheul
Nukta	4	M	Burgers
N’Kato	4	M	Burgers
N’Hasa	4	F	Burgers
*Infants (0–3 years)*			
Madiba	3	M	Burgers
N’Irale	3	F	Burgers

Table 3 shows individuals’ characteristics as a function of name, age (in years), sex (M= male; F= female) and zoo of the study group of gorillas in 2017.

### Data collection

Data were collected by J.P. in 2017 at Apenheul Zoo (June 1st –July 13th ) and at Burgers’ Zoo (July 16th –August 16th ) in the Netherlands, for 77 h respectively 101.5 h (total of 178.5 observation hours). The total observation time per individual ranged between seven and eight hours. Data were collected using a 20 min “focal animal sampling” approach^122^and focusing specifically on subadult individuals only. We tried to observe all subadult individuals for similar duration periods and only focused on the communicative niche of play fighting^51^. Play fighting is defined as the appearance of animals competing in a way that does not look serious and does not lead to the outcomes that are typically associated with the behavior being simulated such as delivering injurious bites or strikes^51^. In subadult gorillas, play-fighting is characterised by behaviours such as play-wrestle and rough-and-tumble play^22^. Observations were made mostly from above and alongside the enclosures to be as close as possible to the study individuals and to ensure the collection of high-quality video data. Data were recorded only when the whole bodies of interactants were visible. Data were collected using a full high-definition video camera (Canon Legria HFM56) equipped with a built-in microphone and a tripod (sampling rule: behaviour sampling; recording rule: continuous recording^123^.

### General coding procedure of social interactions

A total of 1039 high-quality video files including interactions between a subadult and a social partner of any given age class were coded by four coders (J.P., S.W., C.H. and T.F.-M.). We used the Noldus software The Observer XT 14.2^124^to establish the behavioural repertoires of subadults (infants, juveniles and adolescents) used to initiate social play fighting and enable subsequent analyses. Behavioural definitions were based on previously established communication repertoires of gorillas^22,53,125,126^. For the purpose of the present study, we focused the coding on gestures only. Here we define a gesture as a movement of the limbs, head, or body directed towards a recipient that is mechanically ineffective (i.e. “visibly lacks the mechanical force to bring about the reaction shown by the recipient, and also does not include any attempt to grab or extensively hold a body part of the other”^41^p. 8185) and elicits a voluntary response from the recipient^127,128^. A detailed coding scheme was developed based on the parameters described below.

We defined the individual that started the play-fighting interaction as the signaller and the target of this interaction as the recipient. The recipient was identified thanks to the signaller’s intentional behaviours, namely gazing at the recipient, gaze alternation between the enclosure environment and the recipient, body orientation toward the recipient, displacement toward the recipient or physical contact with the recipient in the case of tactile gestures. To ensure statistical independence of data, a gesture was recorded as a new gesture event when a pause in the social interaction or a change in hand activity lasted ≥ 2 s (e.g., the signaller ceased to communicate by leaving the location to search for food sources during 2 s or more). Data were recorded when a gesture was produced singly or in a gesture sequence. A single gesture was defined as an individual producing one gesture, followed by a pause of ≥ 2 s, a direct response of the recipient (e.g., the recipient starts playing), or a change in hand activity of ≥ 2 s. A gesture sequence was defined as an individual producing a series of gestures separated by pauses of < 2 s without a response of the recipient and interspersed periods of response waiting. Only the first gesture of a gesture sequence was taken into account for further analyses, as no evidence was found in^129^for syntactic effects of sequential combination of gestures (separated by pauses of < 1 s) in gorillas. The following conditions had to be met to consider that a single gesture or a gesture sequence was terminated: The signaller’s hand returned to its initial position^94^, switched to another non-communicative activity (e.g., forage, travel), or an incident (e.g., stumble) that could influence the use of one of the hands occurred^130^.

For each dyadic play-fighting interaction, we coded multiple factors and levels related to four key characteristics to investigate determinants of gorillas’ social play fighting focusing on gestural communication: (1) the identity and role of both interactants (signaller and recipient), (2) signaller’s behaviours, (3) gesture characteristics at the stroke phase, and (4) context-related characteristics at the stroke phase (see Table 4 for more details). In total, 20 factors and associated levels were coded to investigate these four key characteristics. In cases where we could not code some levels (particularly subtle gesture characteristics such as flexion and spread of thumb or other fingers) occasionally occurred because of environmental factors (e.g., bushes and ropes) that impaired the coder’s vision. In such cases, we coded “unknown” for these respective level(s).

**Table 4:** List of factors and associated levels considered.

Factors	Levels			
***Interactant identities***				
Signaller’s name	Names of the 16 subadults considered			
Recipient’s name	Names of the 25 other group members (i.e. 15 subadults and ten adults) considered			
***Signaller’s behaviours***				
Gesture type	beat chest / slap ground / slap body / touch body			
Signaller’s body position	lying on front / lying on back / sitting / standing bipedal / standing tripedal / climbing / other body positions			
Signaller’s body motion while standing	walking / running* / no body motion			
***Gesture characteristics at the stroke phase***				
Manuality	unimanual gesture / bimanual gesture			
Manual laterality	left-hand use / right-hand use			
Gesture target	on signaller’s body / towards recipient’s head / towards recipient’s upper body / towards recipient’s lower body / towards an external referent object			
Hand position in relation to signaller’s body	body midline/ between body midline and sides of the body / far from the body			
Horizontal hand trajectory	horizontal plane, from signaller’s body to away / horizontal plane, from away to signaller’s body			
Vertical hand trajectory	vertical plane, from up to down / vertical plane, from down to up			
Main moving body part	trunk joint / shoulder joint / elbow joint / wrist joint / knuckles of the hand			
Physical contact with the recipient	front hand (whole hand with fingers) / front hand (fingers only) / back hand (whole hand with fingers) / back hand (fingers only) / no contact			
Thumb flexion	thumb is stretched / thumb is flexed mid-way towards the palm / thumb is fully flexed towards the palm			
Fingers flexion	other fingers (i.e. index, middle, ring and little fingers) are stretched / other fingers are flexed mid-way / other fingers are fully flexed			
Thumb spread (distance between thumb and index)	thumb is spread outward/ thumb is half spread / thumb and index are bonded			
Fingers spread (distance between fingers)	other fingers (i.e. index, middle, ring and little fingers) are spread outward/ other fingers are half spread / other fingers are bonded			
***Context-related characteristics at the stroke phase***				
Interindividual proximity	body contact / 1 arm / 2 arms / [1–2 m[ / [2–5 m[ / >5 m			
Position of the recipient in the signaller’s visual field	left visual field / right visual field			
Recipient’s attentional state	facing / 90° / >90°			

Table 4 shows a list of all 20 factors and levels we coded for each dyadic play-fighting interaction. * Running is defined as a series of vigorous impulses, resting the body alternately on one then the other foreleg, and at a pace faster than walking. Gesture characteristics are ordered from the most apparent characteristic to the most subtle one.

We only coded behaviours produced during dyadic play interactions with conspecifics characterised by at least one of the following five key traits suggested to study intentional communication in prelinguistic human infants and great apes^18,33,36,131–133^:

1. Use of the signal to achieve a desired social goal as the signal is produced only in the presence of an audience or is produced depending on the size or composition of the audience (e.g., age and sex ratio; kin, affiliative or hierarchical relationship), shown by the signaller’s body or gaze oriented towards a particular recipient or physical contact with a particular recipient;
2. Monitoring of the audience, shown by the signaller looking at a targeted recipient (before, during or shortly after signalling) or alternating its gaze between the recipient and an object or an event;
3. Sensitivity to the attentional state of the recipient, shown by the signaller adjusting the communication in relation to the recipient’s attention (e.g., emitting a visual signal only when the recipient is looking at the signaller or emitting an auditory signal only when the recipient is not looking at the signaller);
4. Waiting for a response, shown by the signaller pausing (for at least 2 s to be consistent with the literature^28,33,134^) while maintaining visual contact with the recipient;
5. Either (i) signaller’s apparent understanding of the recipient’s response, shown by the signaller ceasing to communicate when the initial signal was successful as it had achieved its social goal or when the initial signal was unsuccessful as it did not achieve its social goal because the recipient was not willing to interact or did not understand the signaller or (ii) signaller’s persistence, shown by repetition or elaboration, when the initial signal was unsuccessful (as it did not achieve its social goal).

Before starting the systematic data coding, each of the four observers (J.P., S.W., C.H. and T.F-M.) underwent a training period (about 50 h). During this period, the observers used video recordings of dyadic social-play fighting interactions in subadult gorillas collected by J.P. in 2017 and analysed and coded the behaviours of five focal animals independently with regards to the 20 factors considered in the coding procedure (see Table 4). The data were then compared and discussed with all coders. Training was over when the observations matched in 95% of cases^135^.

#### Coding procedure for signaller’s behaviours

Based on Pika and Bugnyar’s^127^definition of gesture, only behaviours that met the following criteria were classified as gestures (movements of the limbs or head and body): they (a) were used to initiate (i.e. when signaller starts to engage but not continue) a social play interaction, (b) were directed towards a particular recipient as evidenced by signaller’s body or gaze orientation towards the recipient or physical contact with the recipient before or during the performance of the gesture, (c) were mechanically ineffective (i.e. they were not designed to act as direct physical agents), and (d) elicited a voluntary response by recipients (recipients could choose the behavioural outcome in contrast to mechanical effective behaviours when they are physically manipulated by signallers) or no response (i.e. no change in recipient’s gaze and body behaviour). To characterize the gestural production of signallers, we focused on gesture types that have been shown to play a crucial role during social interactions and are produced frequently^12,22,53^: beat chest, slap ground, slap body and touch body (see Table 5).

**Table 5:** Gestural repertoire and detailed description adapted from^22,53,125,136^.

Gesture	Sensory modality	Description					
BEAT CHEST	Auditory	An individual slaps its own chest repetitively by alternating the palms or knuckles (the hand that slapped first is considered to study manual laterality)					
SLAP GROUND	Auditory	An individual hits a surface (usually the ground or a wall or an object) with the palm of one hand.					
SLAP BODY	Tactile	An individual hits the body of a recipient (except genitals) with the palm of one hand without appreciable force. The actual contact with the recipient is more forceful than a brief touch of the recipient’s body.					
TOUCH BODY	Tactile	An individual makes gentle and brief (< 5 s) contact with the recipient’s body (except genitals) with one hand or arm.					

Table 5 shows the investigated gesture types by sensory modality (auditory and tactile) in alphabetical order. Auditory gestures generate a sound while being performed (e.g., SLAP GROUND), while tactile gestures include physical contact with the recipient (e.g., TOUCH BODY)^22^.

#### Coding procedure for gesture characteristics at the stroke phase

**Figure 1:**
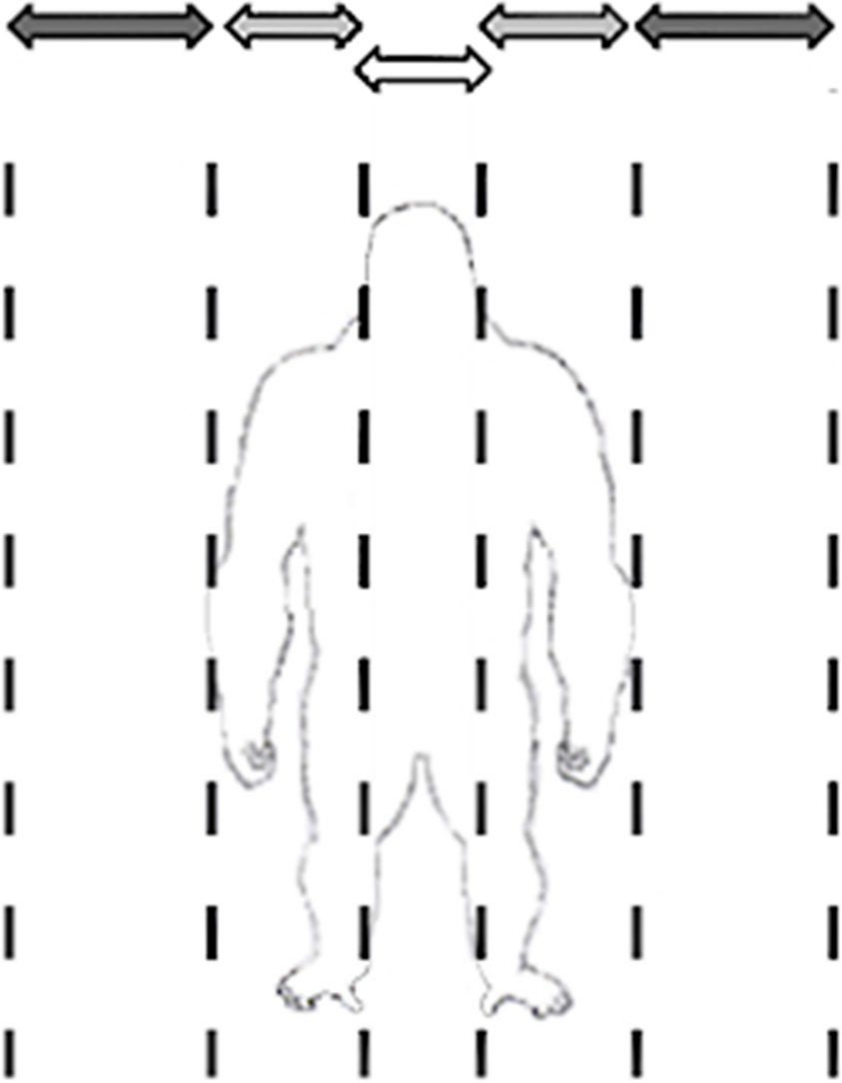
Schematic representation illustrating the three different levels of gesture positions in relation to signaller’s body at the stroke phase (here, the gorilla is represented standing bipedally and before gesturing for clarity). White arrow: body midline; Light grey arrows: between body midline and sides of the body. Dark grey arrows: far from the body.
- ‘Horizontal hand trajectory’ was divided into two levels: horizontal plan, from signaller’s body to away or from away to signaller’s body.
- ‘Vertical hand trajectory’ was divided into two levels: vertical plane, from up to down or from down to up.
- ‘Main moving body part’ was divided into five levels: main movement executed from the trunk joint, the shoulder joint, the elbow joint, the wrist joint or from the hand knuckles.
- ‘Physical contact with the recipient’ was divided into five levels: physical contact with the front/palm of the hand (whole hand and the five fingers or with the five fingers only), with the back of the hand (whole hand and the five fingers or with the five fingers only), or no physical contact with the recipient (i.e. only visual or auditory contact).
- ‘Thumb flexion’ was divided into three levels: stretched thumb, half flexed thumb or fully flexed thumb.
- ‘Fingers flexion’ was divided into three levels: stretched fingers (index, middle, ring and little fingers) (i.e. flat hand), half flexed fingers (i.e. relaxed hand) or fully flexed fingers (i.e. clenched fist).
- ‘Thumb spread’ was divided into three levels: thumb is spread outward, thumb is half spread or thumb and index are bonded.
- ‘Fingers spread’ was divided into three levels: fingers (index, middle, ring and little fingers) are spread outward (i.e. open hand), fingers are half spread (i.e. relaxed hand) or fingers are bonded.

Based on previous studies^43,44,54,55,133^, we investigated the variability of gestural forms and movements by considering twelve gesture characteristics at the stroke phase, which is functionally the most meaningful phase of a gesture^54,55^. The stroke phase happens when the hand physically contacts the target for auditory and tactile gestures (i.e. the signaller’s chest for beat chest, a surface (usually the ground or a wall or an object) for slap ground or the recipient’s body for slap body or touch body). Here are detailed descriptions of each of the twelve gesture characteristics considered (they are ordered from the most apparent characteristic to the subtlest one):

- ‘Manuality’ was divided into two levels: (1) unimanual gestures, which involve the use of only one hand, and (2) bimanual gestures, which involve the use of both hands.
- ‘Manual laterality’ was divided into two levels: left-hand use or right-hand use. Laterality of a given gesture was recorded only during dyadic social interactions satisfying the following two conditions. First, both hands of the signaller were free to communicate and, second, they were symmetrically positioned with respect to its body midline before the interaction, without any environmental factors potentially influencing the use of a specific hand (e.g., being close to a wall/bush/tree^53^.
- ‘Gesture target’ was divided into five levels: towards the recipient’s head, upper body (the part of the body between the neck and the waist (just above the hips)) or lower body (between the hips and the feet), on signaller’s body, and towards an external referent object (e.g., a wooden stick).
- ‘Hand position in relation to signaller’s body’ was divided into three levels: body midline, between body midline and sides of the body or far from the body (Fig. 1).

Three study hand positions in relation to signaller’s body.

#### Coding procedure for context-related characteristics at the stroke phase

Following previous studies^4,35,38,94^, we investigated the relationships between the signaller and its social environment by considering five context-related characteristics at the stroke phase: the signaller’s body position, the signaller’s body motion, the recipient’s attentional state, the position of the recipient in the signaller’s visual field and interindividual proximity. The ‘signaller’s body position’ was divided into seven levels: lying on front, lying on back, sitting, standing bipedal, standing tripedal, climbing and other body positions. The ‘signaller’s body motion’ was divided into three levels: walking, running and no body motion. The ‘recipient’s attentional state’ was divided into three levels: facing (i.e. [-60° to 60°[), 90° (i.e. [60° to 105°[), and > 90° (i.e. [105° to 180°]). The ‘position of the recipient in the signaller’s visual field’ was divided into two levels: left visual field and right visual field. The left visual field is from the direction of the signaller’s head (0°) to the signaller’s left side (180°) whereas the right visual field is from 0° to 180° to the right of the signaller. ‘Interindividual proximity’, defined as the physical distance between both interactants, was divided into six levels: body contact, 1 arm, 2 arms, [1–2 m[, [2–5 m[ and ≥ 5 m.

#### Inter-observer reliability assessment

Over the 662 gestures recorded by the four observers, 10% of the total 526 coded interactions by J.P. (3.3%), S.W. (3.3%) and T.F-M. (3.3%) were coded for accuracy by C.H. and tested using the Cohen’s kappa coefficient to ensure inter-observer reliability^122^. Of the 20 study variables, an ‘almost perfect’ level of agreement (0.81 ≥ κ ≥ 1) was found for eight variables, while a ‘substantial’ level of agreement (0.61 ≥ κ ≥ 0.80) was obtained for nine variables and a ‘moderate’ level of agreement (0.41 ≥ κ ≥ 0.60) was found for “Thumb spread” (see Electronic Supplementary Table 2).

### Sociodemographic characteristics of the individuals

In addition to gorillas’ individual demographic characteristics (i.e. age, sex and location), we took information concerning their genetic relatedness (kin) provided by the two study zoos into account. To assess the potential effect of kinship on gorillas’ social play-fighting behaviour, we considered the following four categories of gorilla pairs according to a threshold coefficient of relatedness:

1. “Parent–infant”: This category included mother–infant and father–infant pairs (coefficient of relatedness (r) = 0.50),
2. “Siblings”: This category included full siblings (r = 0.50),
3. “Half-siblings”: This category included pairs of individuals who share only one biological parent in common (mother or father) (r = 0.25), and
4. “Unrelated”: This category included pairs of genetically unrelated individuals (r < 0.125).

### Statistical analysis

We assessed the effects of signaller’s sociodemographic, signaller’s behavioural and context-related characteristics on gorilla signallers’ gestural forms. Our study’s gesture characteristics are dependent variables with two or more levels. For dependent variables with two levels (i.e. ‘Manuality’, ‘Manual laterality’), we used generalised linear mixed model (GLMM^137^) for binary data (logistic regression) following a logistic regression models approach validated in previous multifactorial studies (e.g^134–139^). For dependent variables with more than two levels (e.g., ‘Gesture target’ with five levels), we followed a standard procedure^140^by splitting the multinomial logistic regression models into a series of binary logistic regression models (e.g., five models to allow comparisons among the five levels of the dependent variable ‘Gesture target’). Signallers’ and recipients’ identities were considered as random variables to prevent pseudo-replication due to repeated observations^141^ (see Electronic Supplementary Table 1). We checked every result provided by the GLMM analysis to detect potential outliers in the odds ratio, standard error of the odds ratio and the z.ratio (see Appendix Table A1).

We used the ‘glmer’ function for GLMM analyses [‘lme4’ package^142^. The best model was the one with the lowest Akaike’s information criterion (AIC). We checked visually equivariance, independence and normality of model residuals using the ‘plotresid’ function [‘RVAideMemoire’ package^143^. The main effects of the best model were tested with type II Wald chi-square tests using the ‘Anova’ function [‘car’ package^144^. Odds ratio were computed using the ‘lsmeans’ function [‘emmeans’ package^145^. Post-hoc multiple comparisons tests were performed using Tukey’s Honest Significant Difference (HSD) test to prevent Type I errors (emmeans package). All statistical analyses were conducted with R version 3.6.3^146^. The level of significance was set at 0.05.

## Supplementary Information

Supplementary Material 1.

Supplementary Material 2.

## Data Availability

The datasets generated during and/or analysed during the current study are available from the corresponding author on reasonable request.
