# Supplementary material for: Social negotiation and “accents” in Western lowland gorillas’ gestural communication
Source: Sci Rep. 2024 Oct 28;14:25699. doi: 10.1038/s41598-024-75238-y (PMC11514168; doi:10.1038/s41598-024-75238-y)
Supplement: Supplementary file 1 — Supplementary Material 1. [file 41598_2024_75238_MOESM1_ESM.docx]

**Electronic Supplementary Table 1. Generalised linear mixed model with dependent, fixed and random variables, their type and associated levels**

| Name | Type |
| --- | --- |
| *Dependent variable* |  |
| Gesture characteristic (see Table 2 for the list of the twelve variables considered) | Dichotomous or multinomial (Table 2) |
| *Fixed variables* |  |
| Signaller's sex | Dichotomous (Female/Male) |
| Signaller's age class | Ordinal (Infant/Juvenile/Adolescent) |
| Recipient's sex | Dichotomous (Female/Male) |
| Recipient's age class | Ordinal (Infant/Juvenile/Adolescent /Young adult/Mature adult) |
| Zoo | Nominal (Apenheul/Burgers) |
| Kinship | Nominal (Parent-infant/Siblings/Half-siblings/Unrelated) |
| Signaller's body position | Nominal (Lying on belly/Lying on back/Sitting/Standing bipedal/Standing tripedal/Climbing/Other body positions) |
| Signaller's body motion while standing | Nominal (Walking/Running/No body motion) |
| Recipient's attentional state | Ordinal (Facing/90°/>90°) |
| Position of recipient in Signaller’s Visual Field (SVF) during interaction | Dichotomous (Left/Right) |
| Interinvidual proximity | Ordinal (Body contact/1 arm/2 arms/[1-2m[/[2-5m[/>5m) |
| *Random variables* |  |
| Signaller's identity | Nominal |
| Recipient's identity | Nominal |

**Electronic Supplementary Table 2. Mean kappa values and associated level of agreement for each study variable**

| **Study variable** | **Mean Kappa** | **Level of agreement** |
| --- | --- | --- |
| Signaller's name | 1 | Almost perfect |
| Recipient's name | 1 |  |
| Gesture target | 1 |  |
| Manual laterality | 0.973 |  |
| Physical contact with the recipient | 0.953 |  |
| Manuality | 0.944 |  |
| Signaller's body position | 0.91 |  |
| Gesture type | 0.9 |  |
| Position of the recipient in the signaller’s visual field | 0.83 |  |
| Interindividual proximity | 0.81 |  |
| Fingers flexion | 0.773 | Substantial |
| Horizontal trajectory | 0.76 |  |
| Signaller's body motion | 0.743 |  |
| Thumb flexion | 0.72 |  |
| Vertical trajectory | 0.72 |  |
| Main moving body part | 0.7 |  |
| Hand position in relation to signaller's body | 0.692 |  |
| Recipient's attentional state | 0.653 |  |
| Fingers spread | 0.63 |  |
| Thumb spread | 0.527 | Moderate |

Electronic Supplementary Table 2 shows the mean kappa values and associated level of agreement [1] for each of the 20 variables considered in the present study. Study variables are presented according to decreasing Mean Kappa values. Mean Kappa: Mean Kappa values between C.H. and each of the three other observers (J.P., S.W. and T.F.-M.). As mentioned by several authors, despite high raw agreement between observers, a relatively low κ value can be an artefact due to low prevalence of a particular parameter [e.g. 2-5]. This is for instance the case for the variable “Fingers spread” (including three modalities: spread outward; half-spread; bonded) which had a high mean level of agreement (77.78%) and a mean Kappa value (κ) equals to 0.63 because “fingers spread outward” were rare: only 9 of the 662 recorded gestures were characterized as “fingers spread outward”. We did not use “Thumb spread” for subsequent statistical analysis because it had a moderate mean level of agreement (58.60%) and a moderate mean Kappa value (κ = 0.527).

**References:**

1. Landis, J. R., & Koch, G. G. (1977). An application of hierarchical kappa-type statistics in the assessment of majority agreement among multiple observers. *Biometrics*, 363-374.
2. Cicchetti, D. V., & Feinstein, A. R. (1990). High agreement but low kappa: II. Resolving the paradoxes. *Journal of clinical epidemiology*, *43*(6), 551-558.
3. Feinstein, A. R., & Cicchetti, D. V. (1990). High agreement but low kappa: I. The problems of two paradoxes. *Journal of clinical epidemiology*, *43*(6), 543-549.
4. Yen, K., Kuppermann, N., Lillis, K., Monroe, D., Borgialli, D., Kerrey, B. T.,…Intra-abdominal Injury study group for the Pediatric Emergency Care applied research Network (PECARN). (2013). Interobserver agreement in the clinical assessment of children with blunt abdominal trauma. Academic Emergency Medicine, 20(5), 426–432.
5. Prieur, J., Le Du, G., Stomp, M., Barbu, S., & Blois-Heulin, C. (2020). Human laterality for manipulation and gestural communication: a study of beach-volleyball players during the Olympic Games. Laterality: Asymmetries of body, Brain and Cognition, 25(2), 229-254.
