# Supplementary material for: Social negotiation and “accents” in Western lowland gorillas’ gestural communication
Source: Sci Rep. 2024 Oct 28;14:25699. doi: 10.1038/s41598-024-75238-y (PMC11514168; doi:10.1038/s41598-024-75238-y)
Supplement: Supplementary file 2 — Supplementary Material 2. [file 41598_2024_75238_MOESM2_ESM.docx]

**Appendix Table A1.**

**Results of post hoc multiple comparisons tests.**

| **Gesture type** | **Dependent variable** | **Fixed variables** | **contrast** |  |  | **odds.ratio** | **SE** | **z.ratio** | ***P*** |  |
| --- | --- | --- | --- | --- | --- | --- | --- | --- | --- | --- |
| Beat chest | Fingers spread (Bonded/Halfspread) | Zoo | Apenheul | - | Burger | 0.092 | 0.079 | -2.762 | **0.006** | |
|  |  | R_position | SVF_L | - | SVF_R | 0.397 | 0.219 | -1.671 | 0.095 | |
|  | Main moving body part (Elbow/Wrist) | R_attention | >90 | - | 90 | 0.382 | 0.283 | -1.300 | 0.395 | |
|  |  |  | >90 | - | facing | 2.541 | 2.151 | 1.102 | 0.513 | |
|  |  |  | 90 | - | facing | 6.645 | 5.187 | 2.427 | **0.040** | |
|  | Manuality (Bimanual/Unimanual) | Posture | lying.on.back | - | sitting | 13.487 | 14.306 | 2.453 | 0.068 | |
|  |  |  | lying.on.back | - | standing.bipedal | 11.855 | 11.346 | 2.584 | **0.048** | |
|  |  |  | lying.on.back | - | standing.tripedal | 2.813 | 3.868 | 0.752 | 0.876 | |
|  |  |  | sitting | - | standing.bipedal | 0.879 | 0.794 | -0.143 | 0.999 | |
|  |  |  | sitting | - | standing.tripedal | 0.209 | 0.290 | -1.127 | 0.673 | |
|  |  |  | standing.bipedal | - | standing.tripedal | 0.237 | 0.305 | -1.119 | 0.678 | |
|  | Manual laterality (Left/Right) | R_sex | F | - | M | 3.970 | 1.740 | 3.141 | **0.002** | |
|  | Vertical hand trajectory  (Downtoup/Uptodown) | Zoo | Apenheul | - | Burger | 5.660 | 2.960 | 3.314 | **0.001** | |
|  |  | R_attention | >90 | - | 90 | 1.850 | 1.044 | 1.090 | 0.520 | |
|  |  |  | >90 | - | facing | 0.481 | 0.240 | -1.465 | 0.308 | |
|  |  |  | 90 | - | facing | 0.260 | 0.137 | -2.552 | **0.029** | |
|  |  | Posture | lying.on.back | - | sitting | 0.959 | 0.789 | -0.051 | 1.000 | |
|  |  |  | lying.on.back | - | standing.bipedal | 9.979 | 7.714 | 2.976 | **0.016** | |
|  |  |  | lying.on.back | - | standing.tripedal | 30.081 | 43.441 | 2.357 | 0.086 | |
|  |  |  | sitting | - | standing.bipedal | 10.410 | 5.643 | 4.322 | **0.0001** | |
|  |  |  | sitting | - | standing.tripedal | 31.379 | 42.239 | 2.560 | 0.051 | |
|  |  |  | standing.bipedal | - | standing.tripedal | 3.014 | 3.841 | 0.866 | 0.823 | |
| Slap body | Manuality (Bimanual/Unimanual) | Zoo | Apenheul | - | Burger | 0.155 | 0.144 | -2.005 | **0.045** | |
|  | Hand location (Betweenbodymidlineandsides/Farfrombody) | R_sex | F | - | M | 0.408 | 0.200 | -1.826 | 0.068 | |

**Appendix Table A1.** continued

| \| **Gesture type** \| **Dependent variable** \| **Fixed variables** \| **contrast** \|  \|  \| **odds.ratio** \| **SE** \| **z.ratio** \| ***P*** \| \| --- \| --- \| --- \| --- \| --- \| --- \| --- \| --- \| --- \| --- \| \| Slap ground \| Manuality (Bimanual/Unimanual) \| Kinship \| Halfsiblings \| - \| Siblings \| 0.132 \| 0.114 \| -2.348 \| **0.050** \| \|  \|  \|  \| Halfsiblings \| - \| Unrelated \| 1.063 \| 0.933 \| 0.069 \| 0.997 \| \|  \|  \|  \| Siblings \| - \| Unrelated \| 8.074 \| 9.077 \| 1.858 \| 0.151 \| \|  \|  \| R_position \| SVF_L \| - \| SVF_R \| 0.393 \| 0.192 \| -1.912 \| 0.056 \| \|  \|  \| Posture \| climbing \| - \| lying.on.back \| 3.611 \| 5.856 \| 0.792 \| 0.986 \| \|  \|  \|  \| climbing \| - \| lying.on.front \| 0.718 \| 1.159 \| -0.205 \| 1.000 \| \|  \|  \|  \| climbing \| - \| other.body.position \| 1.085 \| 1.916 \| 0.046 \| 1.000 \| \|  \|  \|  \| climbing \| - \| sitting \| 2.562 \| 3.930 \| 0.613 \| 0.996 \| \|  \|  \|  \| climbing \| - \| standing.bipedal \| 14.795 \| 22.580 \| 1.765 \| 0.572 \| \|  \|  \|  \| climbing \| - \| standing.tripedal \| 0.073 \| 0.133 \| -1.440 \| 0.780 \| \|  \|  \|  \| lying.on.back \| - \| lying.on.front \| 0.199 \| 0.219 \| -1.466 \| 0.765 \| \|  \|  \|  \| lying.on.back \| - \| other.body.position \| 0.300 \| 0.376 \| -0.961 \| 0.962 \| \|  \|  \|  \| lying.on.back \| - \| sitting \| 0.710 \| 0.609 \| -0.400 \| 1.000 \| \|  \|  \|  \| lying.on.back \| - \| standing.bipedal \| 4.097 \| 3.427 \| 1.686 \| 0.626 \| \|  \|  \|  \| lying.on.back \| - \| standing.tripedal \| 0.020 \| 0.029 \| -2.692 \| 0.100 \| \|  \|  \|  \| lying.on.front \| - \| other.body.position \| 1.509 \| 1.893 \| 0.328 \| 1.000 \| \|  \|  \|  \| lying.on.front \| - \| sitting \| 3.566 \| 3.194 \| 1.419 \| 0.792 \| \|  \|  \|  \| lying.on.front \| - \| standing.bipedal \| 20.591 \| 18.651 \| 3.340 \| **0.015** \| \|  \|  \|  \| lying.on.front \| - \| standing.tripedal \| 0.102 \| 0.137 \| -1.704 \| 0.614 \| \|  \|  \|  \| other.body.position \| - \| sitting \| 2.362 \| 2.618 \| 0.776 \| 0.987 \| \|  \|  \|  \| other.body.position \| - \| standing.bipedal \| 13.641 \| 14.953 \| 2.384 \| 0.205 \| \|  \|  \|  \| other.body.position \| - \| standing.tripedal \| 0.068 \| 0.100 \| -1.819 \| 0.535 \| \|  \|  \|  \| sitting \| - \| standing.bipedal \| 5.774 \| 3.627 \| 2.792 \| **0.077** \| \|  \|  \|  \| sitting \| - \| standing.tripedal \| 0.029 \| 0.036 \| -2.794 \| 0.077 \| \|  \|  \|  \| standing.bipedal \| - \| standing.tripedal \| 0.005 \| 0.006 \| -4.127 \| **0.001** \| \|  \| Manual laterality (Left/Right) \| R_attention \| >90 \| - \| 90 \| 2.853 \| 1.636 \| 1.828 \| 0.160 \| \|  \|  \|  \| >90 \| - \| facing \| 0.979 \| 0.467 \| -0.044 \| 0.999 \| \|  \|  \|  \| 90 \| - \| facing \| 0.343 \| 0.160 \| -2.296 \| 0.056 \| \|  \| Hand location (Betweenbodymidlineandsides/Farfrombody) \| R_sex \| F \| - \| M \| 0.272 \| 0.152 \| -2.234 \| 0.020 \| \|  \|  \| R_attention \| >90 \| - \| 90 \| 0.468 \| 0.448 \| -0.794 \| 0.707 \| \|  \|  \|  \| >90 \| - \| facing \| 0.180 \| 0.146 \| -2.119 \| 0.086 \| \|  \|  \|  \| 90 \| - \| facing \| 0.385 \| 0.257 \| -1.431 \| 0.325 \| |  |  |  |  |  |  |  |  |  |
| --- | --- | --- | --- | --- | --- | --- | --- | --- | --- | --- | --- | --- | --- | --- | --- | --- | --- | --- | --- | --- | --- | --- | --- | --- | --- | --- | --- | --- | --- | --- | --- | --- | --- | --- | --- | --- | --- | --- | --- | --- | --- | --- | --- | --- | --- | --- | --- | --- | --- | --- | --- | --- | --- | --- | --- | --- | --- | --- | --- | --- | --- | --- | --- | --- | --- | --- | --- | --- | --- | --- | --- | --- | --- | --- | --- | --- | --- | --- | --- | --- | --- | --- | --- | --- | --- | --- | --- | --- | --- | --- | --- | --- | --- | --- | --- | --- | --- | --- | --- | --- | --- | --- | --- | --- | --- | --- | --- | --- | --- | --- | --- | --- | --- | --- | --- | --- | --- | --- | --- | --- | --- | --- | --- | --- | --- | --- | --- | --- | --- | --- | --- | --- | --- | --- | --- | --- | --- | --- | --- | --- | --- | --- | --- | --- | --- | --- | --- | --- | --- | --- | --- | --- | --- | --- | --- | --- | --- | --- | --- | --- | --- | --- | --- | --- | --- | --- | --- | --- | --- | --- | --- | --- | --- | --- | --- | --- | --- | --- | --- | --- | --- | --- | --- | --- | --- | --- | --- | --- | --- | --- | --- | --- | --- | --- | --- | --- | --- | --- | --- | --- | --- | --- | --- | --- | --- | --- | --- | --- | --- | --- | --- | --- | --- | --- | --- | --- | --- | --- | --- | --- | --- | --- | --- | --- | --- | --- | --- | --- | --- | --- | --- | --- | --- | --- | --- | --- | --- | --- | --- | --- | --- | --- | --- | --- | --- | --- | --- | --- | --- | --- | --- | --- | --- | --- | --- | --- | --- | --- | --- | --- | --- | --- | --- | --- | --- | --- | --- | --- | --- | --- | --- | --- | --- | --- | --- | --- | --- | --- | --- | --- | --- | --- | --- | --- | --- | --- | --- | --- | --- | --- | --- | --- | --- | --- | --- | --- | --- | --- | --- | --- | --- | --- | --- | --- | --- | --- | --- | --- | --- | --- | --- | --- | --- | --- | --- | --- | --- | --- | --- | --- | --- | --- | --- | --- | --- | --- | --- | --- | --- | --- | --- | --- | --- | --- | --- | --- | --- | --- | --- |

**Appendix Table A1.** continued

| \| **Gesture type** \| **Dependent variable** \| **Fixed variables** \| **contrast** \|  \|  \| **odds.ratio** \| **SE** \| **z.ratio** \| ***P*** \| \| --- \| --- \| --- \| --- \| --- \| --- \| --- \| --- \| --- \| --- \| \| Slap ground \| Vertical hand trajectory  (Downtoup/Uptodown) \| Zoo \| Apenheul \| - \| Burger \| 19.600 \| 14.700 \| 3.977 \| **0.0001** \| \|  \|  \| Posture \| climbing \| - \| lying.on.back \| 1.851 \| 2.860 \| 0.398 \| 1.000 \| \|  \|  \|  \| climbing \| - \| lying.on.front \| 0.345 \| 0.582 \| -0.630 \| 0.996 \| \|  \|  \|  \| climbing \| - \| other.body.position \| 31.424 \| 50.219 \| 2.157 \| 0.319 \| \|  \|  \|  \| climbing \| - \| sitting \| 0.883 \| 1.252 \| -0.088 \| 1.000 \| \|  \|  \|  \| climbing \| - \| standing.bipedal \| 2.875 \| 3.863 \| 0.786 \| 0.986 \| \|  \|  \|  \| climbing \| - \| standing.tripedal \| 1.293 \| 1.987 \| 0.167 \| 1.000 \| \|  \|  \|  \| lying.on.back \| - \| lying.on.front \| 0.187 \| 0.247 \| -1.266 \| 0.867 \| \|  \|  \|  \| lying.on.back \| - \| other.body.position \| 16.980 \| 20.199 \| 2.381 \| 0.207 \| \|  \|  \|  \| lying.on.back \| - \| sitting \| 0.477 \| 0.454 \| -0.778 \| 0.987 \| \|  \|  \|  \| lying.on.back \| - \| standing.bipedal \| 1.554 \| 1.341 \| 0.510 \| 0.999 \| \|  \|  \|  \| lying.on.back \| - \| standing.tripedal \| 0.699 \| 0.720 \| -0.348 \| 1.000 \| \|  \|  \|  \| lying.on.front \| - \| other.body.position \| 90.986 \| 128.633 \| 3.191 \| **0.024** \| \|  \|  \|  \| lying.on.front \| - \| sitting \| 2.557 \| 3.071 \| 0.782 \| 0.987 \| \|  \|  \|  \| lying.on.front \| - \| standing.bipedal \| 8.324 \| 9.457 \| 1.865 \| 0.504 \| \|  \|  \|  \| lying.on.front \| - \| standing.tripedal \| 3.744 \| 4.895 \| 1.010 \| 0.952 \| \|  \|  \|  \| other.body.position \| - \| sitting \| 0.028 \| 0.029 \| -3.419 \| **0.011** \| \|  \|  \|  \| other.body.position \| - \| standing.bipedal \| 0.092 \| 0.085 \| -2.585 \| 0.130 \| \|  \|  \|  \| other.body.position \| - \| standing.tripedal \| 0.041 \| 0.048 \| -2.766 \| 0.083 \| \|  \|  \|  \| sitting \| - \| standing.bipedal \| 3.255 \| 2.123 \| 1.809 \| 0.542 \| \|  \|  \|  \| sitting \| - \| standing.tripedal \| 1.464 \| 1.339 \| 0.417 \| 1.000 \| \|  \|  \|  \| standing.bipedal \| - \| standing.tripedal \| 0.450 \| 0.375 \| -0.960 \| 0.962 \| \| Touch body \| Manual laterality (Left/Right) \| R_attention \| >90 \| - \| 90 \| 4.294 \| 2.756 \| 2.271 \| 0.060 \| \|  \|  \|  \| >90 \| - \| facing \| 1.197 \| 0.633 \| 0.340 \| 0.938 \| \|  \|  \|  \| 90 \| - \| facing \| 0.279 \| 0.144 \| -2.476 \| **0.035** \| \|  \|  \| R_position \| SVF_L \| - \| SVF_R \| 0.495 \| 0.203 \| -1.712 \| 0.087 \| \|  \| Gesture target (Head/Lowerbody) \| R_attention \| >90 \| - \| 90 \| 2.710 \| 2.300 \| 1.172 \| 0.470 \| \|  \|  \|  \| >90 \| - \| facing \| 10.670 \| 7.890 \| 3.202 \| **0.004** \| \|  \|  \|  \| 90 \| - \| facing \| 3.940 \| 3.020 \| 1.790 \| 0.173 \| \|  \| Gesture target (Lowerbody /Upperbody) \| R_attention \| >90 \| - \| 90 \| 0.288 \| 0.180 \| -1.996 \| 0.113 \| \|  \|  \|  \| >90 \| - \| facing \| 0.185 \| 0.104 \| -2.999 \| **0.008** \| \|  \|  \|  \| 90 \| - \| facing \| 0.642 \| 0.383 \| -0.743 \| 0.738 \| |  |  |  |  |  |  |  |  |  |
| --- | --- | --- | --- | --- | --- | --- | --- | --- | --- | --- | --- | --- | --- | --- | --- | --- | --- | --- | --- | --- | --- | --- | --- | --- | --- | --- | --- | --- | --- | --- | --- | --- | --- | --- | --- | --- | --- | --- | --- | --- | --- | --- | --- | --- | --- | --- | --- | --- | --- | --- | --- | --- | --- | --- | --- | --- | --- | --- | --- | --- | --- | --- | --- | --- | --- | --- | --- | --- | --- | --- | --- | --- | --- | --- | --- | --- | --- | --- | --- | --- | --- | --- | --- | --- | --- | --- | --- | --- | --- | --- | --- | --- | --- | --- | --- | --- | --- | --- | --- | --- | --- | --- | --- | --- | --- | --- | --- | --- | --- | --- | --- | --- | --- | --- | --- | --- | --- | --- | --- | --- | --- | --- | --- | --- | --- | --- | --- | --- | --- | --- | --- | --- | --- | --- | --- | --- | --- | --- | --- | --- | --- | --- | --- | --- | --- | --- | --- | --- | --- | --- | --- | --- | --- | --- | --- | --- | --- | --- | --- | --- | --- | --- | --- | --- | --- | --- | --- | --- | --- | --- | --- | --- | --- | --- | --- | --- | --- | --- | --- | --- | --- | --- | --- | --- | --- | --- | --- | --- | --- | --- | --- | --- | --- | --- | --- | --- | --- | --- | --- | --- | --- | --- | --- | --- | --- | --- | --- | --- | --- | --- | --- | --- | --- | --- | --- | --- | --- | --- | --- | --- | --- | --- | --- | --- | --- | --- | --- | --- | --- | --- | --- | --- | --- | --- | --- | --- | --- | --- | --- | --- | --- | --- | --- | --- | --- | --- | --- | --- | --- | --- | --- | --- | --- | --- | --- | --- | --- | --- | --- | --- | --- | --- | --- | --- | --- | --- | --- | --- | --- | --- | --- | --- | --- | --- | --- | --- | --- | --- | --- | --- | --- | --- | --- | --- | --- | --- | --- | --- | --- | --- | --- | --- | --- | --- | --- | --- | --- | --- | --- | --- | --- | --- | --- | --- | --- | --- | --- | --- | --- | --- | --- | --- | --- | --- | --- | --- | --- | --- | --- | --- | --- | --- | --- | --- | --- | --- | --- | --- | --- | --- | --- | --- | --- | --- | --- | --- | --- | --- | --- |

R: Recipient; S: Signaller; SVF_L: Left signaller’s visual field side; SVF_R: Right signaller’s visual field side; odds.ratio: odds ratio value; SE: standard error of the odds ratio; z.ratio: ratio of the odds ratio to its standard error; *P*: Tukey’s p-value. Significant results are in bold.
